# Supplementary material for: Transcriptional dynamics of murine motor neuron maturation in vivo and in vitro
Source: Nat Commun. 2022 Sep 15;13:5427. doi: 10.1038/s41467-022-33022-4 (PMC9477853; doi:10.1038/s41467-022-33022-4)
Supplement: Supplementary file 1 — Supplementary Information [file 41467_2022_33022_MOESM1_ESM.pdf]

Supplementary Figure 1

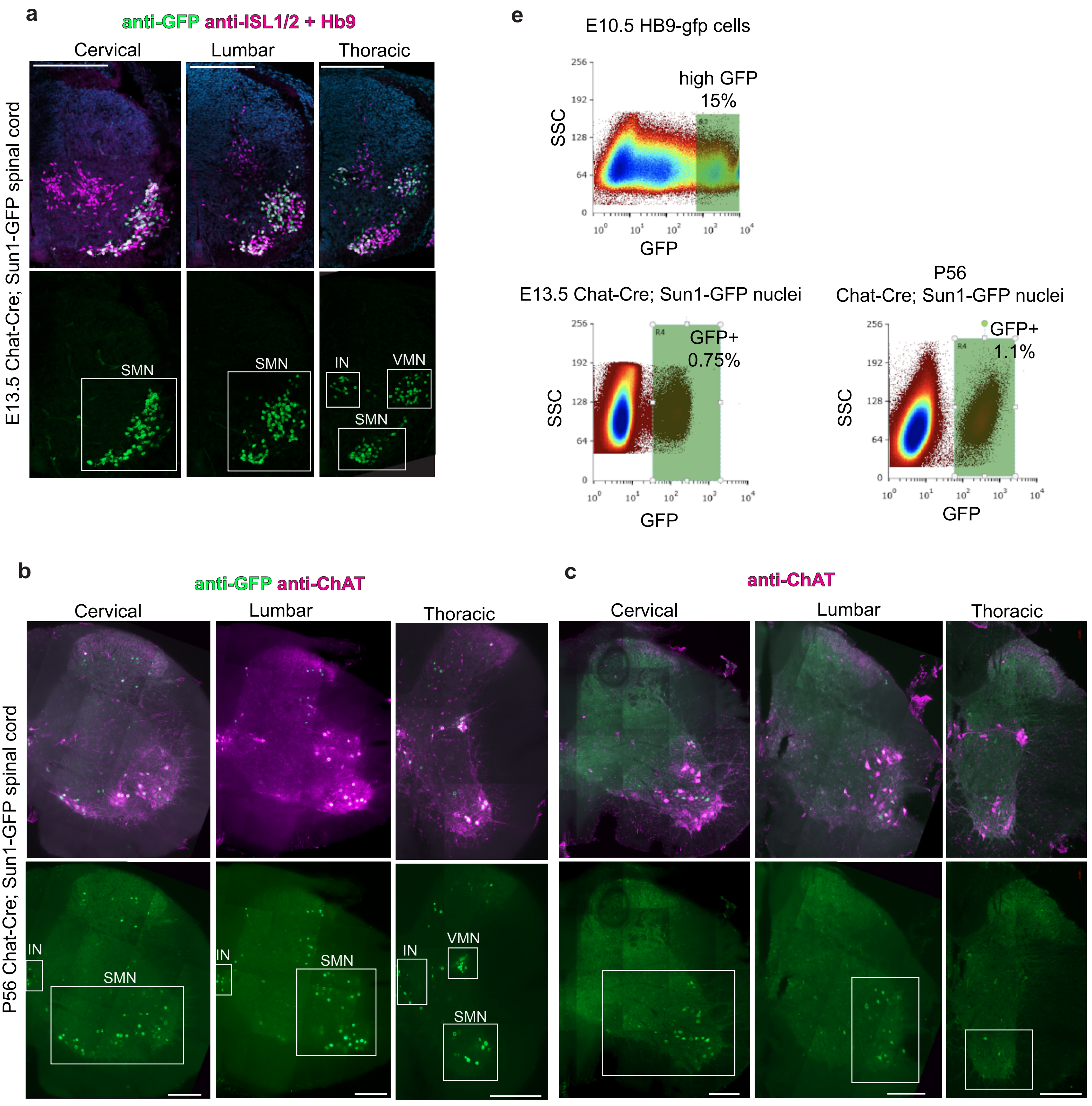

d For RNA-seq and ATAC-seq (E10.5): FACs used to isolate whole cells from Cervical +Thoracic + Lumbar cords
For RNA-seq (E13.5 - 2yr): INTACT used to isolate nuclei from Cervical + Lumbar cords
For ATAC-seq (E13.5 - 2yr): FACs used to isolate nuclei from Cervical + Thoracic + Lumbar cords

Supplementary Figure 1: Labeling and purification of motor neurons. a) Spinal cord sections from E13.5 Chat-Cre; Sun1-GFP mice stained for GFP and motor neuron transcription factors ISL1/2 and HB9 (magenta). **b,c**) Spinal cord sections from P56 Chat-Cre; Sun1-GFP mice stained for GFP and cholinergic gene ChAT (magenta) (b) or just ChAT (c). Motor neurons show highest endogenous expression of SUN1-GFP in the absence of anti-GFP immunostaining. Labels indicate regions of the spinal cord where cholinergic skeletal motor neurons (SMN), interneurons (IN) and visceral motor neurons (VMN) are found. **d**) Spinal cord segments and method used to purify nuclei for RNA-seq and ATAC-seq. **e**) FACs parameters used for sorting GFP+ cells or nuclei. All scale bars are 200 um.

Supplementary Figure 2

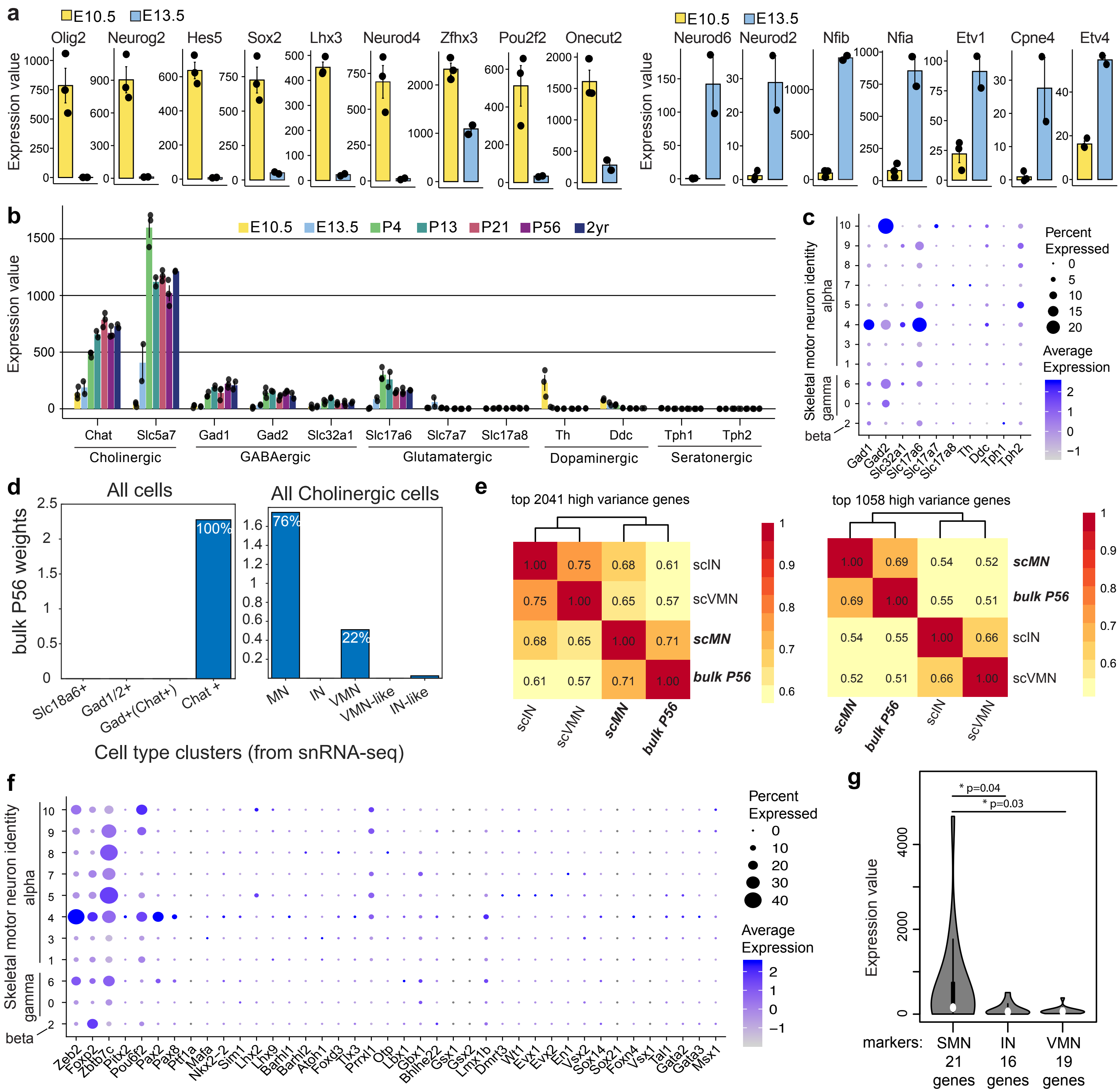

Supplementary Figure 2: Bulk RNA-seq data is enriched for skeletal motor neurons. a) Expression values of genes previously shown to be downregulated between E10.5 and E13.5 (left panel) or upregulated between E10.5 and E13.5 (right panel). Error bars show SEM; n = 3, 2 (E10.5, E13.5) biological replicates, shown as black dots. **b,c**) Expression of neurotransmitter pathway genes in bulk RNA-seq data generated in this study (b) and in single cell analysis of skeletal motor neurons performed by Blum et al., 2021(c). In (b) error bars show SEM; n = 3 biological replicates for all expect E13.5 and P13 (n = 2), shown as black dots. **d**) Deconvolution of bulk P56 RNA-seq data into snRNA-seq clusters from Alkaslasi et al., 2021. The weights on the y-axis denote representation of each cell type cluster in the bulk dataset. Left panel includes all cholinergic and non-cholinergic cells in snRNA-seq dataset, right panel includes only cholinergic cells. **e**) Pearson correlation between bulk gene expression at P56 and single cell expression in cholinergic skeletal motor neurons (SMN), visceral motor neurons (VMN) and interneurons (IN) (snseq data from integrated Blum et al., 2021 and Alkaslasi et al., 2021). Correlation is performed on the top 2041 and 1058 genes that show high variance between cholinergic SMN, VMN, and IN. **f**) Dot plot showing expression of VMN and IN genes from Fig. 1d in motor neurons. Motor neurons expression data comes from snRNA-seq performed by Blum et al., 2021. Genes such as *Zeb2, Foxp2* are expressed in both VMN/IN and subsets of motor neurons. **g**) Expression of SMN, IN, and VMN terminal effectors genes (identified in Blum et al., and Alkaslasi et al., 2021) in bulk P56 RNA-seq data generated in this study. Two-tailed t-test, n = 3 biological replicates. For boxplots center line is the median, the interquartile range is 25th percentile-75th percentile; p-values are determined by two-tailed t-tests. Source data are provided as a Source Data file.

Supplementary Figure 3

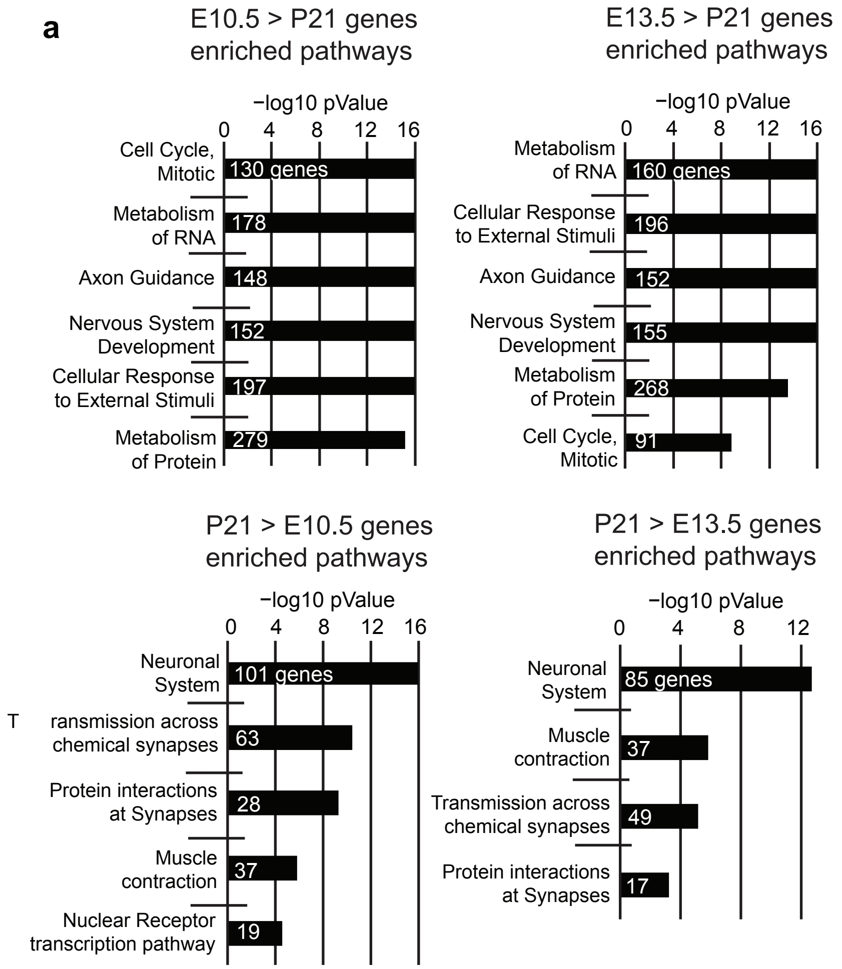

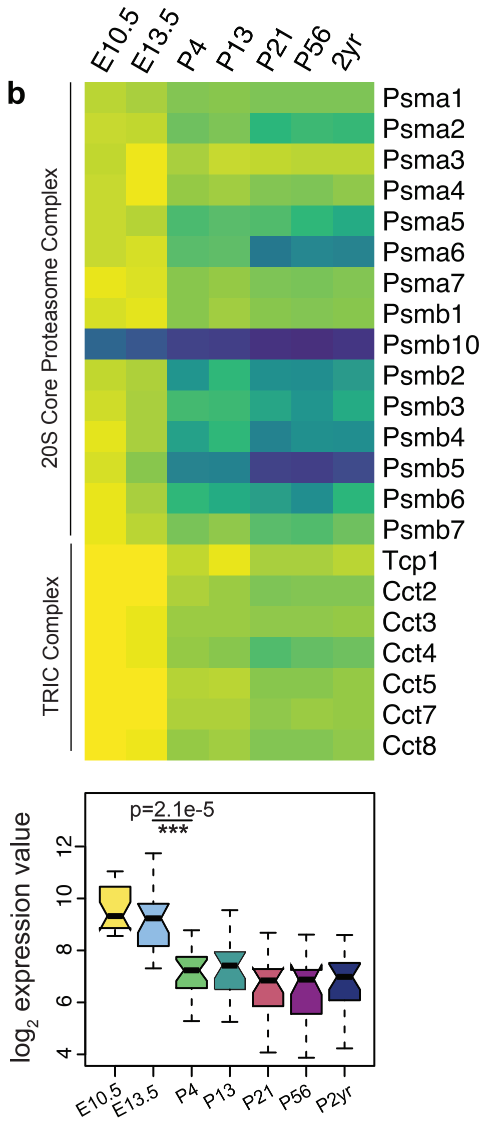

Supplementary Figure 3: Gene expression changes during motor neuron maturation. a) The most significant pathways enriched in the top 1000 genes up- and downregulated during maturation between E10.5 and P21, or between E13.5 and P21. P-values generated by Reactome. **b)** Expression of proteasome complex genes during maturation. For boxplots center line is the median, the interquartile range is 25th percentile-75th percentile, and outliers are eliminated; p-values are determined by two-tailed t-tests.

Supplementary Figure 4

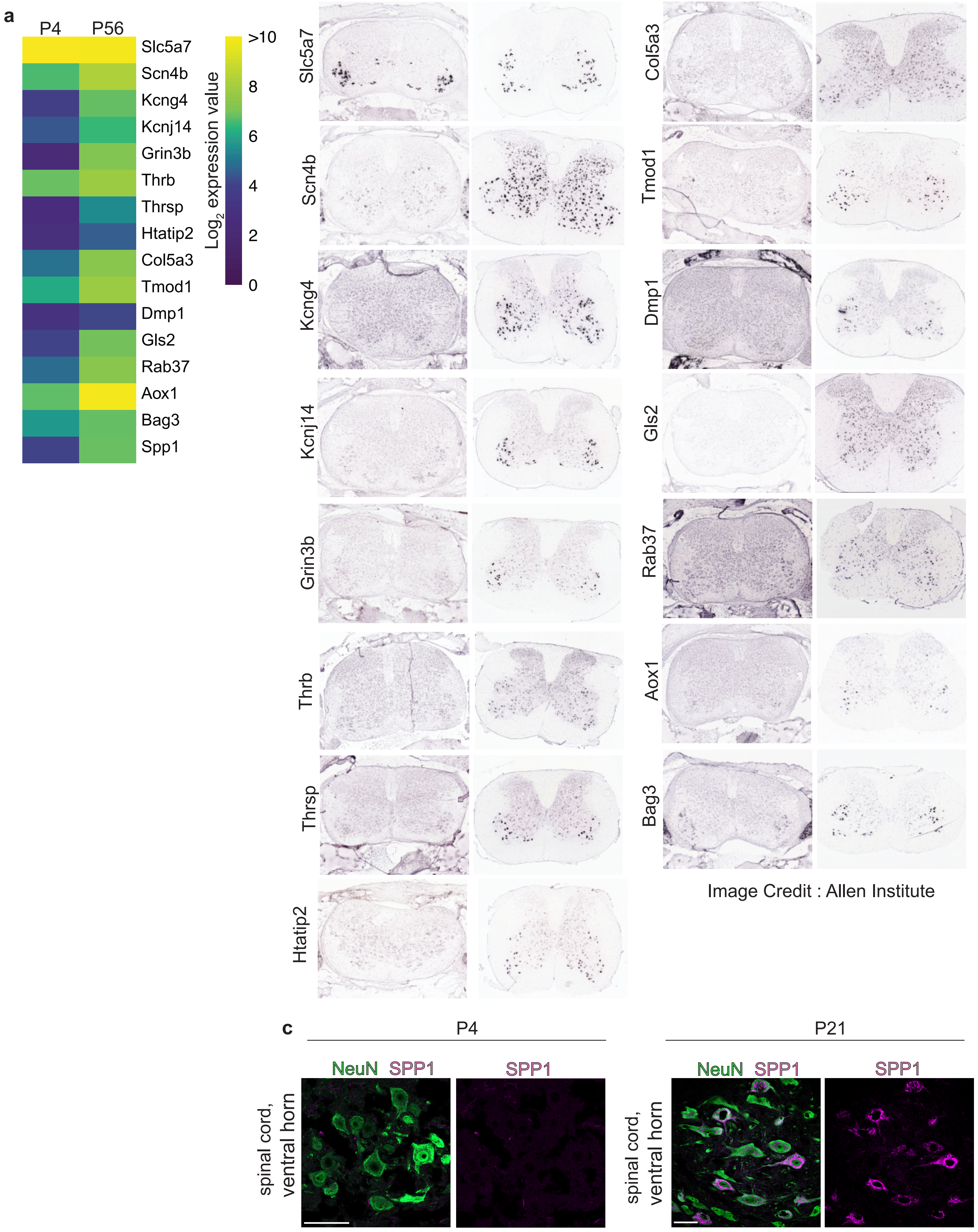

Supplementary Figure 4: Validation of genes upregulated during motor neuron maturation. a) Heat map showing expression of a continuously expressed gene, *Slc5a7*, and 13 genes that are upregulated over time. **b**) Allen Spinal Cord Atlas in situ hybridization images of genes from (a) at P4 and P56. **c**) Immunostaining of Spp1 in P4 motor neurons and P21 motor neurons. Image shows the ventral horn of the spinal cord. Scale bars are 50 um.

Supplementary Figure 5

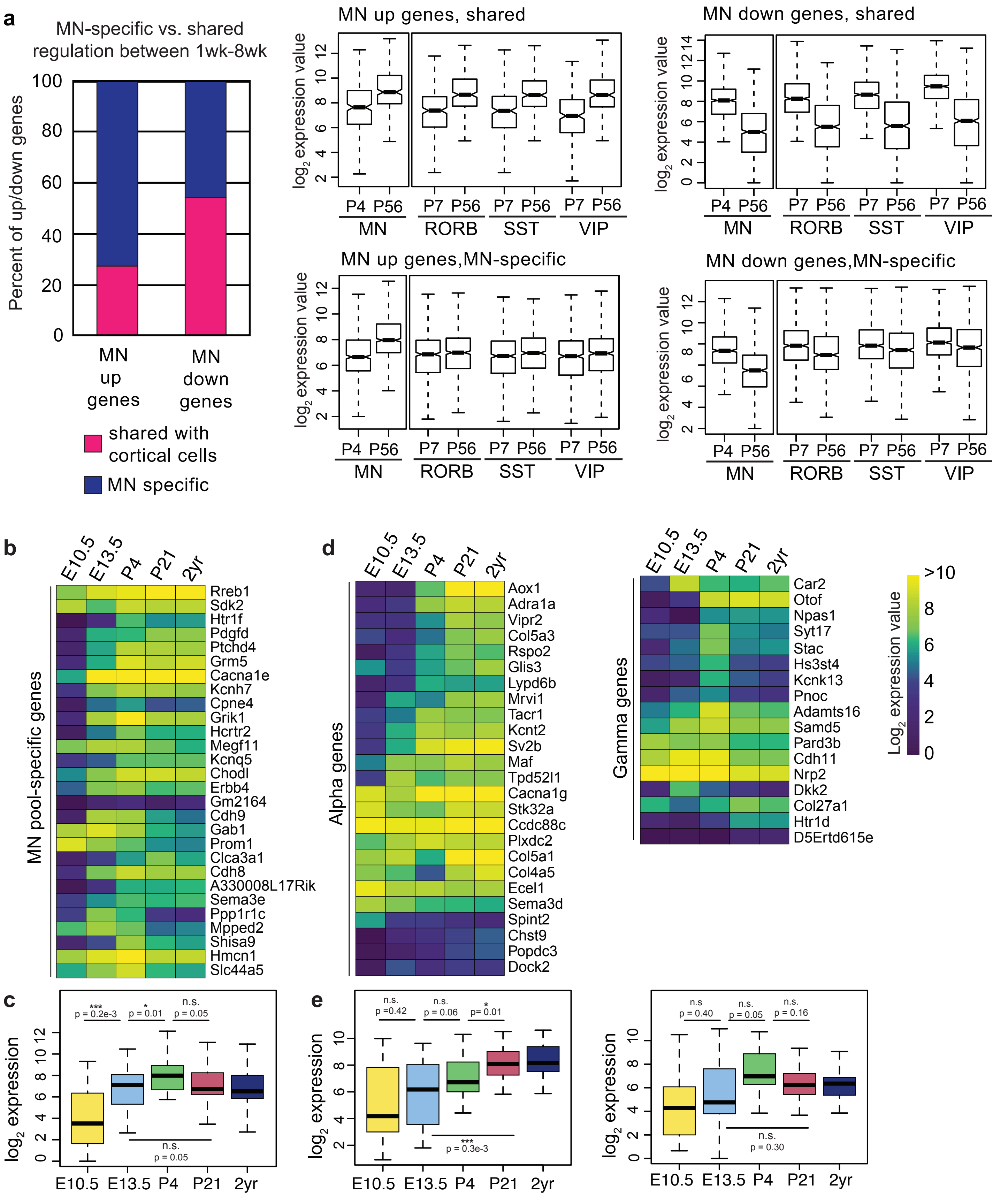

Supplementary Figure 5: Cell type specificity of genes upregulated during motor neuron maturation. a) Percent of motor neuron up- and downregulated genes that are either shared with VIP, SST, and RORB neurons, or not, and the $\log_2$ expression values of shared and specific gene sets. For motor neurons, gene expression between P56 and P4 timepoints are compared. For VIP, SST, and RORB neurons, gene expression between P7 and P56 timepoints are compared. N= 276 shared up, 948 shared down, 728 specific up, and 794 specific down genes. **b-e**) Heatmaps and boxplots showing expression of motor pool specific genes, alpha, and gamma genes. Lists of marker genes were obtained from Blum et al., 2021 and Alkaslasi et al., 2021. Boxplots only include genes that are detected postnatally. For all boxplots, center line is the median, the interquartile range is 25th percentile-75th percentile, and outliers are eliminated. Two-tailed t-tests, * $p< 0.05$, *** $p<0.0005$.

Supplementary Figure 6

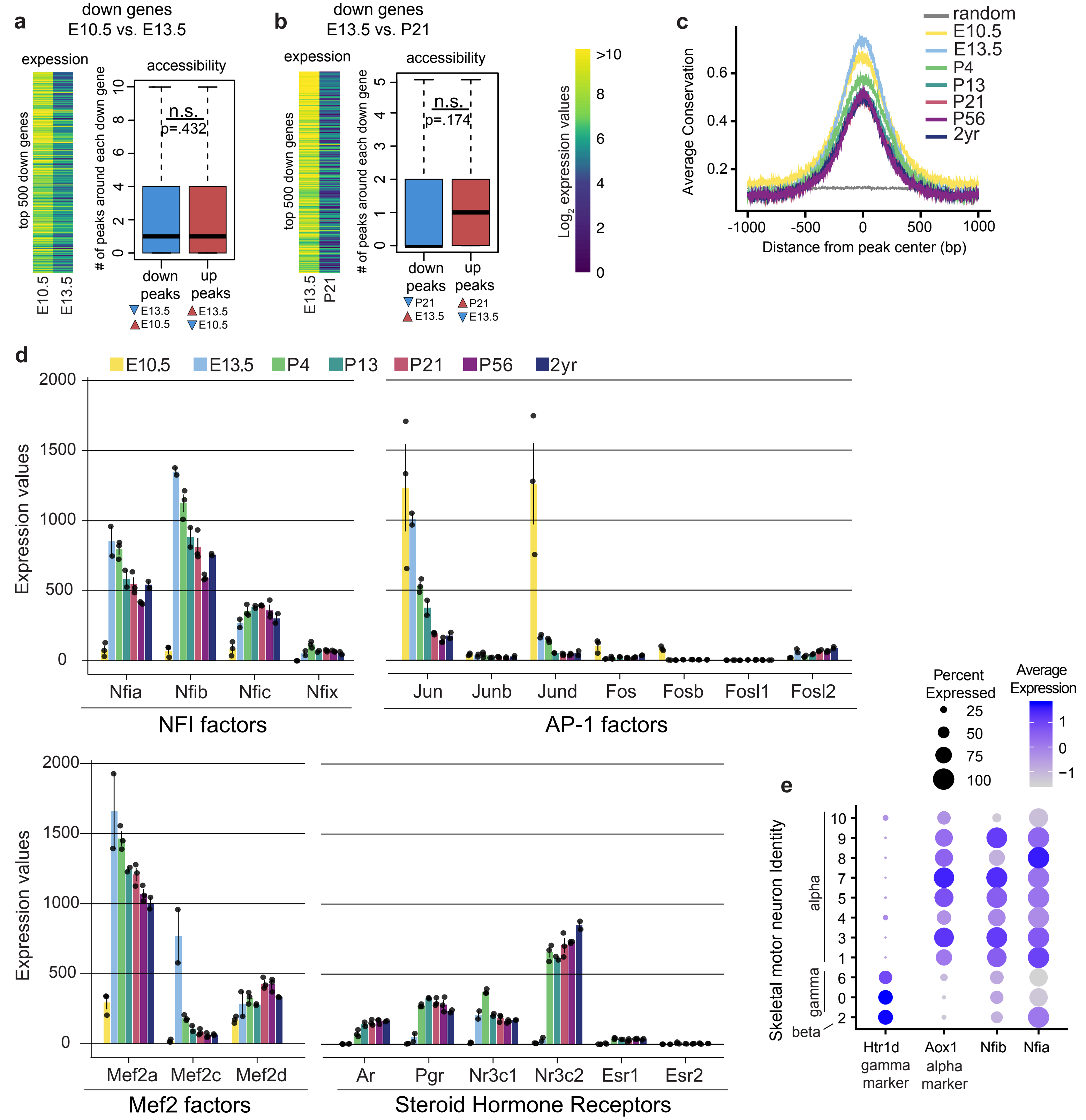

Supplementary Figure 6: Identification of candidate regulators from ATAC-seq data. a, b) The distribution of differentially accessible regions near genes that are downregulated during maturation between E10.5-E13.5 and E13.5-P21. The gene expression heat maps show the expression values of top 500 downregulated genes. The boxplots show the number of peaks that become newly accessible (red) or lose accessibility (blue) between E10.5-E13.5 and E13.5-P21 around each of the 500 downregulated genes. For all boxplots, center line is the median, the interquartile range is 25th percentile-75th percentile, and outliers are eliminated; two-tailed t-tests; n = 500 genes. **c**) Average conservation of top 10k motor neuron accessible regions at each age compared to random genomic sequences. **d**) Expression values of all transcription factors belonging to NFI, AP-1, Mef2, and steroid hormone receptors families at all profiled ages. Error bars show SEM; n = 3 biological replicates for all expect E13.5 and P13 (n = 2), shown as black dots. **e**) Dot plot showing expression of *Nfia* and *Nfib* in motor neuron snRNA-seq data performed by Blum et al., 2021. Source data are provided as a Source Data file.

Supplementary Figure 7

Top 5 de novo motifs at each age (repeated motifs were manually eliminated)

HOMER output format

de novo motif:
match:

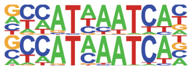

	E10.5 (p-value / **%targets** / %backgroud)	E13.5 (p-value / **%targets** / %backgroud)	P4 (p-value / **%targets** / %backgroud)	P13 (p-value / **%targets** / %backgroud)	P21 (p-value / **%targets** / %backgroud)	P56 (p-value / **%targets** / %backgroud)	2yr (p-value / **%targets** / %backgroud)
Hox	1e-594 / **35.15%** / 14.24%	1e-892 / **44.48%** / 16.97%	1e-502 / **31.65%** / 13.09%	1e-480 / **30.21%** / 12.42%	1e-317 / **38.61%** / 21.74%		1e-299 / **34.32%** / 18.67%
RXR	1e-138 / **9.18%** / 3.62%						
Sox	1e-513 / **52.01%** / 28.83%						
E-Box	1e-336 / **41.19%** / 23.44%						
LIM	1e-236 / **39.58%** / 24.66%	1e-405 / **58.01%** / 36.73%	1e-135 / **56.84%** / 44.45%				
Onecut		1e-195 / **39.12%** / 25.48%					
NFI		1e-440 / **28.47%** / 11.79%	1e-141 / **49.25%** / 36.82%				
NFI half			1e-179 / **39.95%** / 26.75%	1e-227 / **61.01%** / 44.94%	1e-242 / **50.27%** / 34.04%	1e-241 / **53.11%** / 36.76%	1e-204 / **38.91%** / 25.03%
CRE		1e-167 / **21.41%** / 11.66%	1e-201 / **18.67%** / 8.90%	1e-161 / **14.61%** / 6.82%	1e-166 / **17.33%** / 8.65%	1e-544 / **35.85%** / 15.43%	1e-804 / **33.43%** / 10.72%
GRE half				1e-115 / **34.61%** / 24.38%	1e-166 / **58.76%** / 45.00%	1e-185 / **46.06%** / 32.09%	1e-186 / **53.46%** / 39.02%
COE						1e-186 / **42.39%** / 28.70%	1e-164 / **46.66%** / 33.41%
AP-1					1e-608 / **27.29%** / 9.00%		
Mef2				1e-165 / **8.61%** / 2.93%		1e-172 / **11.29%** / 4.45%	

matched to Meox2 by HOMER

matched to Zfp691 by HOMER

Motifs were manually assinged to LIM and GRE families even though HOMER default match was different

Supplementary Figure 7: Motifs enriched at each age. HOMER outputs of top 5 *de novo* motifs enriched at each age. The *de novo* motifs identified (top in all motif logos), as well as the match to known transcription factor family binding motifs (bottom in all motif logos) are shown. The p-value and prevalence of enriched motif, as determined by HOMER, is reported on the right of each logo. In two instances (blue and red boxes), *de novo* motifs were matched manually rather than using the default HOMER match.

Supplementary Figure 8

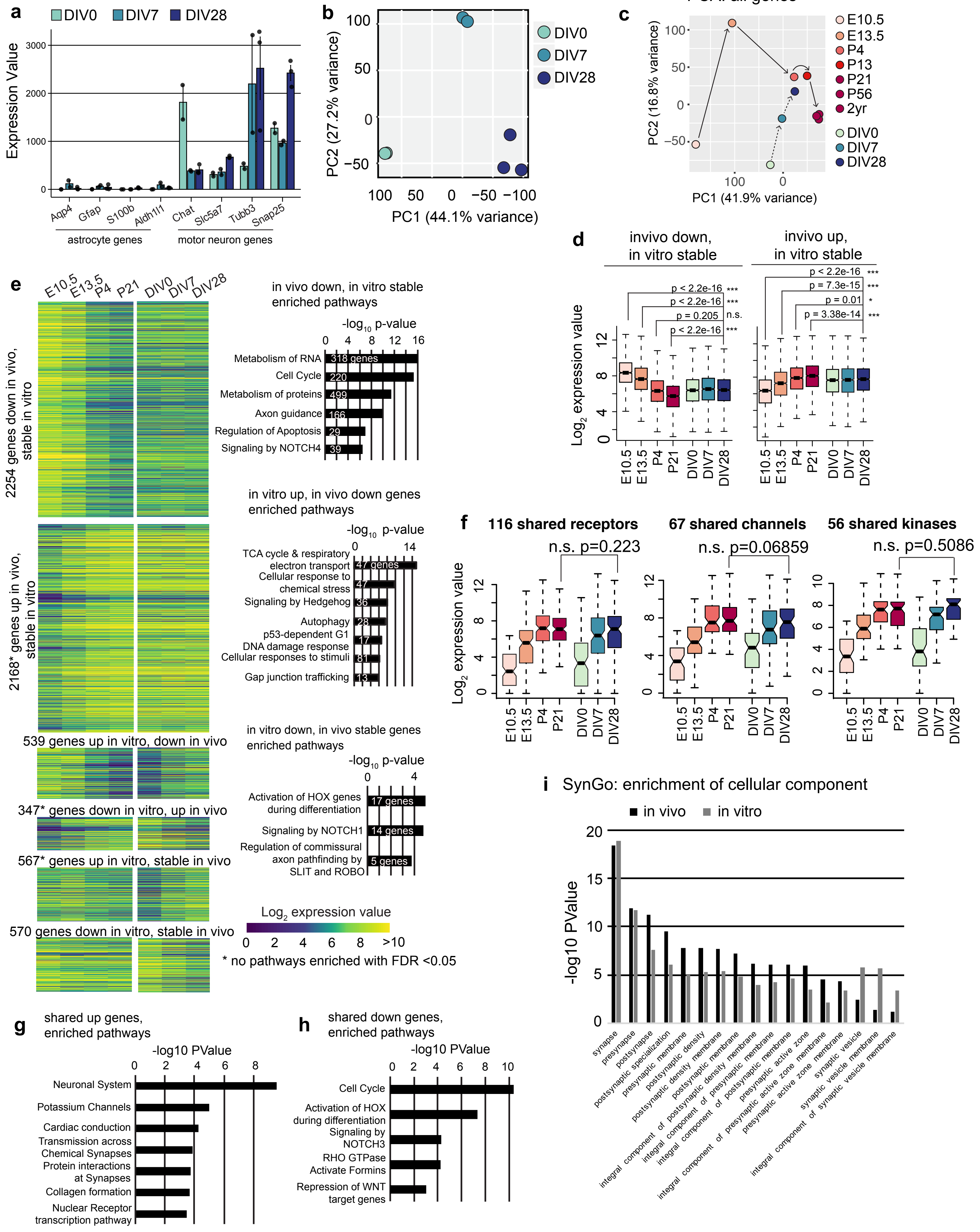

Supplementary Figure 8: Comparison of gene expression changes *in vitro* and *in vivo*. a) Expression levels of astrocyte genes and motor neuron genes in in vitro RNA-seq data. Error bars show SEM; n = 2 (DIV0, DIV7), 3 (DIV28) biological replicates, shown as black dots. **b)** Principal Component Analysis on *in vitro* RNA-seq data at DIV0, DIV7, DIV28. Circles of the same color represent biological replicates for each time point. **c**) Principal Component Analysis on all expressed genes at all *in vivo* and *in vitro* timepoints. Each dot is the average expression of all biological replicates. **d**) Boxplots showing expression of 2255 and 2176 genes that are downregulated or upregulated, respectively, *in vivo* but remain stable *in vitro*. P-values are determined by two-tailed t-tests. **e**) Heatmaps on left show the *in vivo* and *in vitro* expression trajectories of all categories of genes in Fig. 4g. Barplots on right show the most enriched pathways in specified gene sets with p-values determined by Reactome. **f**) Boxplots show expression of terminal effectors genes that are upregulated during *in vivo* and *in vitro* maturation. P-values are determined by two-tailed t-tests. **g,h**) The most significant pathways enriched in genes that are similarly regulated *in vitro* and *in vivo*. **i**) Categories of synaptic genes that are enriched in the *in vitro* or *in vivo* maturation programs. For all boxplots in this figure, center line is the median, the interquartile range is 25th percentile-75th percentile, and outliers are eliminated; p-values are determined by two-tailed t-tests. Source data are provided as a Source Data file.

Supplementary Figure 9

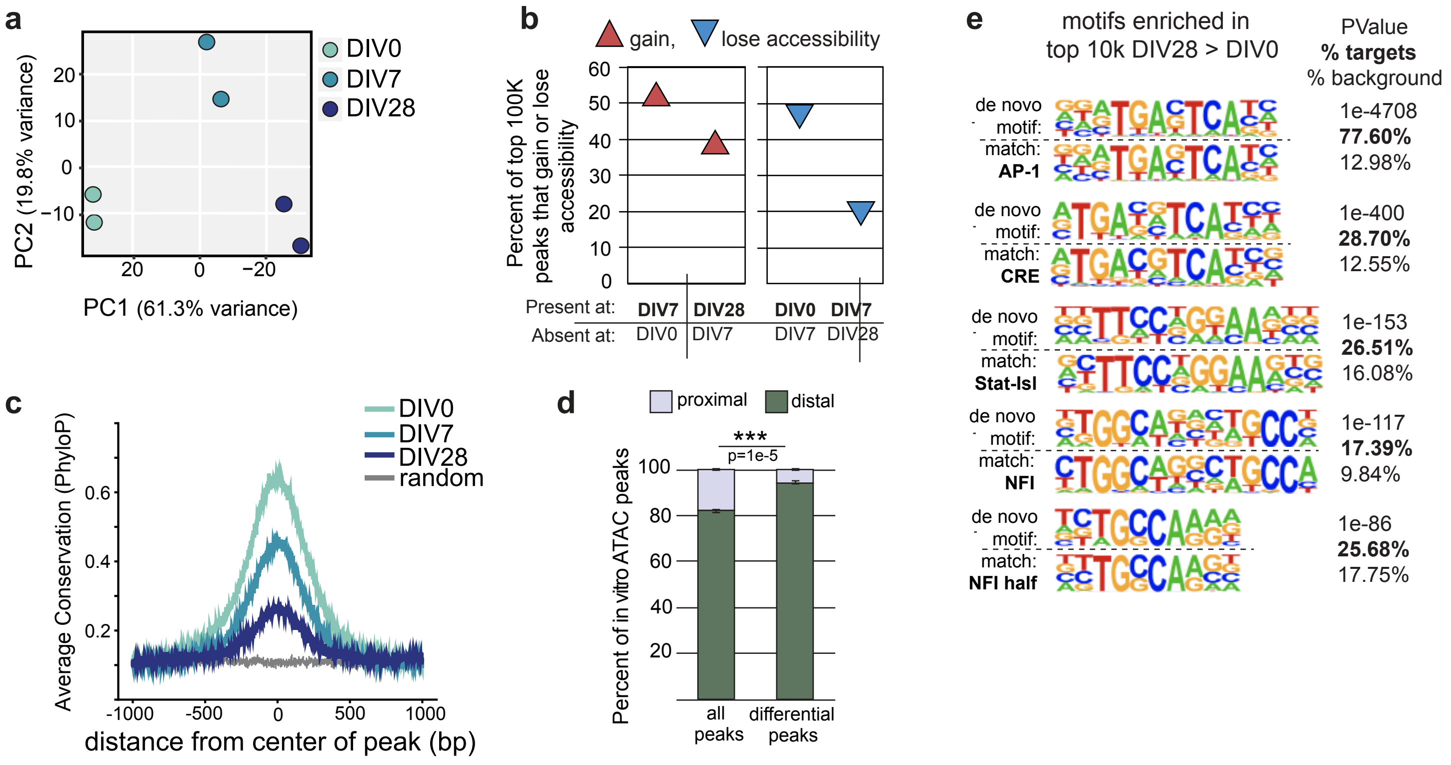

Supplementary Figure 9: Identifying regulators of *in vivo* and *in vitro* maturation. a) Principal Component Analysis on ATAC-seq data at DIV0, DIV7, and DIV28. Circles of the same color represent biological replicates. **b**) The percent of top 100k genomic regions that lose accessibility or become newly accessible between consecutive timepoints. **c**) The conservation of *in vitro* motor neuron accessible regions at each time point compared to random genomic sequences. **d**) The percent of total peaks or differential peaks that are proximal (within 2kb) or distal (>2kb away) from transcription start sites. Error bars are SEM; n = 3 for all peaks, n = 4 for differential peaks, two-tailed t-tests. **e**) Top motifs enriched in top 10k DIV28 > DIV0 peaks. The motif logos show the *de novo* motif identified by HOMER on top and the best matched known transcription factor motif on the bottom. The p-value and prevalence of enriched motif, as determined by HOMER, is reported on the right.

Supplementary Figure 10

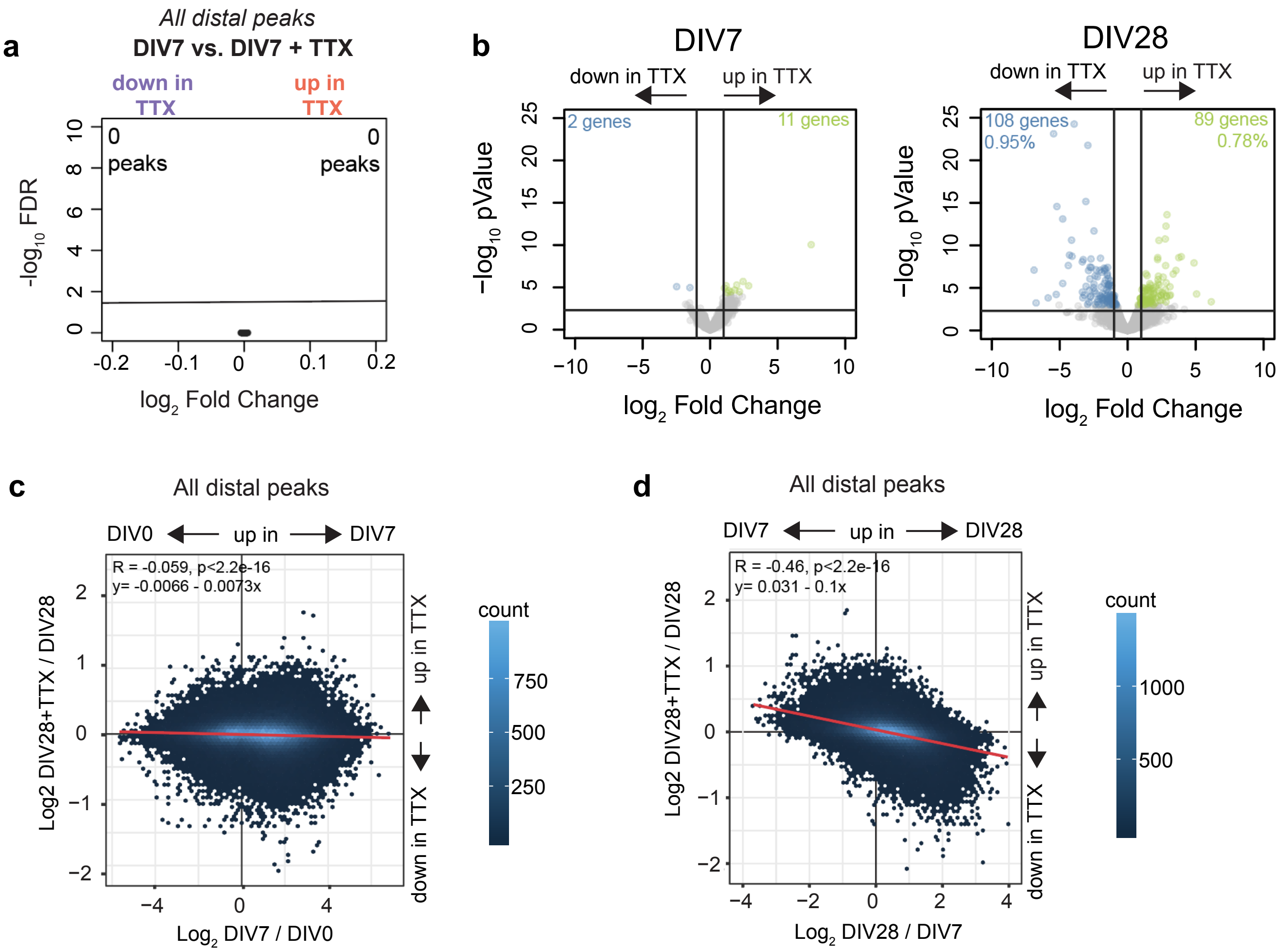

Supplementary Figure 10: Effect of activity on maturation-dependent changes in chromatin accessibility and gene expression. a) Differential accessibility analysis between DIV7 and DIV7 + TTX conditions. Each dot represents a single ATAC-seq peak. No peaks gain or lose accessibility with FDR < 0.05, as calculated by DiffBind. **b)** Plots showing differential gene expression between DIV7 and DIV7 + TTX (left) and DIV28 and DIV28 + TTX (right) conditions. Each dot represents one gene, the x-axis shows the $\log_2$ fold change, the y-axis shows the $-\log_{10}$ p-value. Colored dots are genes that are upregulated (green) or downregulated (blue) at least 2-fold with a p-value < 0.001 (as determined by EdgeR). **c**) A density map that plots the correlation in global accessibility changes between DIV0-DIV7 compared to accessibility changes due to loss of activity. Each hexagon on the plot represents a number of ATAC-peaks (as shown in the scale on the right), the x-axis plots the change in accessibility over time, and y-axis plots the change in accessibility in DIV28 + TTX condition compared to DIV28. For example the bottom right quadrant contains peaks that are normally upregulated in culture between DIV0-DIV7, but are down regulated in the absence of activity. **d**) Same as (c) for time interval between DIV7-DIV28.
