# Supplementary material for: Transcriptional dynamics of murine motor neuron maturation in vivo and in vitro
Source: Nat Commun. 2022 Sep 15;13:5427. doi: 10.1038/s41467-022-33022-4 (PMC9477853; doi:10.1038/s41467-022-33022-4)
Supplement: Supplementary file 6 — Reporting Summary [file 41467_2022_33022_MOESM6_ESM.pdf]

nature portfolio

Corresponding author(s): Hynek Wichterle
Tulsi Patel

Last updated by author(s): Jul 31, 2022

Reporting Summary

Nature Portfolio wishes to improve the reproducibility of the work that we publish. This form provides structure for consistency and transparency in reporting. For further information on Nature Portfolio policies, see our Editorial Policies and the Editorial Policy Checklist.

Statistics

For all statistical analyses, confirm that the following items are present in the figure legend, table legend, main text, or Methods section.

n/a	Confirmed	
☐	☒	The exact sample size (n) for each experimental group/condition, given as a discrete number and unit of measurement
☒	☐	A statement on whether measurements were taken from distinct samples or whether the same sample was measured repeatedly
☐	☒	The statistical test(s) used AND whether they are one- or two-sided<br>*Only common tests should be described solely by name; describe more complex techniques in the Methods section.*
☒	☐	A description of all covariates tested
☒	☐	A description of any assumptions or corrections, such as tests of normality and adjustment for multiple comparisons
☐	☒	A full description of the statistical parameters including central tendency (e.g. means) or other basic estimates (e.g. regression coefficient) AND variation (e.g. standard deviation) or associated estimates of uncertainty (e.g. confidence intervals)
☐	☒	For null hypothesis testing, the test statistic (e.g. F, t, r) with confidence intervals, effect sizes, degrees of freedom and P value noted<br>*Give P values as exact values whenever suitable.*
☒	☐	For Bayesian analysis, information on the choice of priors and Markov chain Monte Carlo settings
☒	☐	For hierarchical and complex designs, identification of the appropriate level for tests and full reporting of outcomes
☒	☐	Estimates of effect sizes (e.g. Cohen's d, Pearson's r), indicating how they were calculated

Our web collection on statistics for biologists contains articles on many of the points above.

Software and code

Policy information about availability of computer code

Data collection	All collection of genomic data was performed with previously published software and packages listed here (versions provided when relevant): Cutadapt v0.6.2; RSEM (v1.3.0); STAR (v2.5.2b); Python (v3.6.9); EdgeR (multiple versions); bwa mem (v0.7.1.7) ; samtools (v1.7.2); MACS2 (v2.2.7.1); BEDTools; DiffBind; R; Matlab. Details in Methods of manuscript. Code used in this study is available on GitHub [https://github.com/gifford-lab/motor-neuron-maturation]
Data analysis	All analysis of genomic data was performed with previously published software and packages listed here (versions provided when relevant): Cutadapt v0.6.2; RSEM (v1.3.0); STAR (v2.5.2b); Python (v3.6.9); EdgeR (multiple versions); bwa mem (v0.7.1.7) ; samtools (v1.7.2); MACS2 (v2.2.7.1); BEDTools; DiffBind; R; Matlab. Details in Methods of manuscript. Code used in this study is available on GitHub [https://github.com/gifford-lab/motor-neuron-maturation]

For manuscripts utilizing custom algorithms or software that are central to the research but not yet described in published literature, software must be made available to editors and reviewers. We strongly encourage code deposition in a community repository (e.g. GitHub). See the Nature Portfolio guidelines for submitting code & software for further information.

Data

Policy information about availability of data

All manuscripts must include a data availability statement. This statement should provide the following information, where applicable:

- Accession codes, unique identifiers, or web links for publicly available datasets
- A description of any restrictions on data availability
- For clinical datasets or third party data, please ensure that the statement adheres to our policy

All sequencing data generated in this study have been deposited to GEO database under accession code GSE198767 [https://www.ncbi.nlm.nih.gov/geo/query/acc.cgi?acc=GSE198767]

Field-specific reporting

Please select the one below that is the best fit for your research. If you are not sure, read the appropriate sections before making your selection.

[x] Life sciences [ ] Behavioural & social sciences [ ] Ecological, evolutionary & environmental sciences

For a reference copy of the document with all sections, see nature.com/documents/nr-reporting-summary-flat.pdf

Life sciences study design

All studies must disclose on these points even when the disclosure is negative.

Sample size	Sample size was not predetermined. Sample sizes were chosen based on common convention in the field (for example, Stroud et al., Neuron 2020). 2-3 biological replicates were performed for all in vivo sequencing experiments with n=3-9 animals of mixed sex, and atleast three replicates for in vitro experiments. Over 100 cells were scored (from atleast 3 replicates) for results generated from immunostaining studies. Sample sizes for experiments are provided in figure captions and methods.
Data exclusions	No data is excluded from this study.
Replication	Experiments were verified by replication to ensure consistency and reliability of data. A conventional number of replicates were used depending on experiment type (for example, Stroud et al., Neuron 2020). 2-3 biological replicates were performed for all in vivo sequencing experiments with n=3-9 animals of mixed sex, and at least three replicates for in vitro experiments.
Randomization	Data randomization was used to subset random numbers of cells from snseq data for the bulk RNA-seq data deconvolution
Blinding	Blinding was not used in this study, but scoring of images was performed by two independent investigators.

Reporting for specific materials, systems and methods

We require information from authors about some types of materials, experimental systems and methods used in many studies. Here, indicate whether each material, system or method listed is relevant to your study. If you are not sure if a list item applies to your research, read the appropriate section before selecting a response.

Materials & experimental systems

n/a	Involved in the study	
[ ]	[x]	Antibodies
[ ]	[x]	Eukaryotic cell lines
[x]	[ ]	Palaeontology and archaeology
[ ]	[x]	Animals and other organisms
[x]	[ ]	Human research participants
[x]	[ ]	Clinical data
[x]	[ ]	Dual use research of concern

Methods

n/a	Involved in the study	
[x]	[ ]	ChIP-seq
[x]	[ ]	Flow cytometry
[x]	[ ]	MRI-based neuroimaging

Antibodies

Antibodies used	The following primary antibodies were used: GFP (Chick, Thermo Fisher A10262, 1:3000), Chat (Goat, EMD Millipore AB144P 1:100), hb9 (Guinea pig 1:100 from Jessell Lab), NeuN (Rabbit, Millipore Sigma ABN78, 1:1000), Spp1 (Mouse, R&D Systems AF808, 1:50; Goat, sc-21742, 1:300), Isl1/2 (Ms 4D5 DSHB optimized concentration by staining; Neuromics GT15051 1:5000); Nfia (Rb, Active Motif 39397, 1:1000), Nfib (Rb, Active Motif 39091, 1:1000), NFH (Chk, Neuromics CH22104, 1:2000), cFos (Rat, Synaptic Systems 226 017, 1:500), Nr3c1 (Rabbit, Invitrogen PA1-511A, 1:2000), Nr3c2 (DSHB clones 1D5 and 3F10, 1:33). The following secondary antibodies were used: 1:800, 706-165-148, 711-165-152, 715-165-151, 715-545-150, 712-605-153, 712-605-152, 712-605-151, 703-545-155,

	715-175-151, 715-175-150 from Jackson ImmunoResearch Laboratories.
Validation	We used commonly used antibodies. Examples of publications validating antibodies: Spp1- Morisaki et al., Scientific Reports volume 6, Article number: 27354 (2016); NFI - Chen et al., 2017, JCN; Nr3c2- McCann et al. 2021, Molecular Psychiatry; Neun, Hb9 - Jacko et al., Neuron, 2018.

Eukaryotic cell lines

Policy information about cell lines

Cell line source(s)	Generation of new mESC lines is described in Methods.
Authentication	All lines were verified by genotyping and sequencing of multiple independent clones.
Mycoplasma contamination	All lines were tested regularly to verify lack of mycoplasma contamination.
Commonly misidentified lines (See ICLAC register)	NA

Animals and other organisms

Policy information about studies involving animals; ARRIVE guidelines recommended for reporting animal research

Laboratory animals	Mice were used in accordance with IACUC guidelines and protocols approved by the Institute of Comparative Medicine at Columbia University. The mice are housed in microisolator cages in a pathogen-free barrier<br>facility. All animals will are under daily surveillance by the staff and veterinarians of the animal facility and<br>the Institute of Comparative Medicine at Columbia University. The specific pathogen-free status of the colony<br>is monitored using sentinel mice.
Wild animals	No wild animals were used in this study.
Field-collected samples	No field-collected samples were used in this study.
Ethics oversight	Protocols are approved by Institutional Animal Care and Use Committee (IACUC) at the Columbia University Institute of Comparative Medicine . The animal care and use program at Columbia University is accredited by the AAALAC International and maintains an Animal Welfare Assurance with the Public Health Service (PHS), Assurance number D16-00003 (A3007-01).

Note that full information on the approval of the study protocol must also be provided in the manuscript.
